# Antiplatelet Agents Have a Distinct Efficacy on Platelet Aggregation Induced by Infectious Bacteria

**DOI:** 10.3389/fphar.2020.00863

**Published:** 2020-06-05

**Authors:** Nadji Hannachi, Emma Ogé-Ganaye, Jean-Pierre Baudoin, Anthony Fontanini, Denis Bernot, Gilbert Habib, Laurence Camoin-Jau

**Affiliations:** ^1^Aix Marseille Univ, IRD, APHM, MEPHI, IHU Méditerranée infection, Marseille, France; ^2^Laboratoire d’Hématologie, Hôpital de la Timone, APHM, Boulevard Jean-Moulin, Marseille, France; ^3^Département de cardiologie, Hôpital de la Timone, AP-HM, Boulevard Jean-Moulin, Marseille, France

**Keywords:** aggregation, antiplatelet agents, platelets, *Staphylococcus aureus*, *Streptococcus sanguinis*

## Abstract

Platelets are the cornerstone of hemostasis. However, their exaggerated aggregation induces deleterious consequences. In several diseases, such as infectious endocarditis and sepsis, the interaction between platelets and bacteria leads to platelet aggregation. Despite platelet involvement, no antiplatelet therapy is currently recommended in these infectious diseases. We aimed here, to evaluate, *in vitro*, the effect of antiplatelet drugs on platelet aggregation induced by two of the bacterial pathogens most involved in infectious endocarditis, *Staphylococcus aureus* and *Streptococcus sanguinis*. Blood samples were collected from healthy donors (n = 43). Treated platelet rich plasmas were incubated with three bacterial strains of each species tested. Platelet aggregation was evaluated by Light Transmission Aggregometry. CD62P surface exposure was evaluated by flow cytometry. Aggregate organizations were analyzed by scanning electron microscopy. All the strains tested induced a strong platelet aggregation. Antiplatelet drugs showed distinct effects depending on the bacterial species involved with different magnitude between strains of the same species. Ticagrelor exhibited the highest inhibitory effect on platelet activation (p <0.001) and aggregation (p <0.01) induced by *S. aureus*. In the case of *S. sanguinis*, platelet activation and aggregation were better inhibited using the combination of both aspirin and ticagrelor (p <0.05 and p <0.001 respectively). Aggregates ultrastructure and effect of antiplatelet drugs observed by scanning electron microscopy depended on the species involved. Our results highlighted that the effect of antiplatelet drugs depended on the bacterial species involved. We might recommend therefore to consider the germ involved before introduction of an optimal antiplatelet therapy.

## Introduction

Platelet–bacteria interactions are a cornerstone of several infectious vascular damages such as Disseminated Intra-vascular Coagulation (DIC) following sepsis or embolic events following infectious endocarditis (IE) (Hamzeh-Cognasse et al., 2015).

Staphylococci and streptococci represent the most incriminated germs in IE (Park et al., 2016; Habib et al., 2019). The presence of protein receptors at the surface of these bacteria allows them to interact with platelets thereby promoting their aggregation (Ford et al., 1993; Hamzeh-Cognasse et al., 2015; Hannachi et al., 2019b). These platelet–bacteria interactions seem to be diverted in favor of bacteria despite the recognized immune role of platelets. Indeed, platelets would provide a platform for bacterial nesting in the case of IE. In addition, the formation of aggregates around the bacteria allows their protection against host immunity as well as their dissemination through bloodstream (Kahn et al., 2013; Paharik and Horswill, 2016).

While antiplatelet agents largely demonstrated their benefit in the management of cardiovascular diseases, they are not proposed in the management of hemostatic events particularly induced by infectious bacteria (Chan et al., 2003; Eikelboom et al., 2012). Indeed, the studies already carried out have not reached a conclusive result yet (Ford et al., 1993; Arman et al., 2014; Veloso et al., 2015; Chabert et al., 2017). Ford et al. reported that aspirin decreased aggregation induced by *S. sanguinis* (Ford et al., 1993). Arman et al. reported that cangrelor and indomethacin had differential effect according to the bacterial species involved (Arman et al., 2014). In contrast, Chabert et al. reported no aggregation induced by *S. aureus* (Chabert et al., 2017).

We have reported in a recent study carried out on aspirin that the latter showed distinct effects on platelet aggregation according to the bacterial species involved (Hannachi et al., 2019a). In this current study, we aimed to evaluate all types of oral antiplatelet drugs used currently in clinical practice on platelet aggregation induced by different strains of each bacterial species (*S. aureus* and *S. sanguinis*), looking for possible inter species and inter-strain variability. Our study was based on functional methods (Light Transmission Aggregometry) and phenotypic analyzes (flow cytometry and scanning electron microscopy).

## Material and Methods

### Platelet Preparation

Blood was drawn by venipuncture into sodium citrate from healthy subjects without any medication (n = 43). Platelet-Rich Plasma (PRP) was prepared according to the ISTH recommendation (Cattaneo et al., 2013). Briefly, after keeping the blood at rest for 15 min, sample was centrifuged at 200×*g* for 10 min at ambient temperature without using a brake. Platelet count determination was performed using a hematology analyzer. Platelet count was adjusted using platelet poor plasma (PPP) to get 2.5 × 10^8^ platelet/ml. Then, PRP was treated by aspirin (Sanofi, Toulouse, France) at a final concentration of 2 mM (Laudy et al., 2016; Dotto et al., 2017), a concentration relatively high compared to circulating peak reached *in vivo*, in order to totally acetylate platelet cyclooxygenase, or by ticagrelor (AstraZeneca AB S-151 85 Södertälje, Sweden) at a final concentration of 10 μM (Söderlund et al., 2015) or by the combination of the two drugs, or by tirofiban (Agrastat, United Kingdom) at a final concentration of 0.5 μM (Ciborowski et al., 2008). A part of PRP remained untreated. For flow cytometry, treated and untreated PRP were centrifuged at 1,100*g* for 10 min to get platelet pellet that was resuspended in Tyrode’s buffer to obtain 2.5 × 10^8^ p/ml. The protocol was approved by the ethic committee of the IHU Méditerranée-infection (Reference 2016-002).

### Bacterial Preparation

Strains from the CSUR (Collection des souches de l’unité des Rickettsies, IHU Méditerranée infection, Marseille France) were used. Bacterial strains were identified by Maldi Toff mass spectrometry using the Bio Typer database (Bruker, Dresden, Germany). In a second time, they were cultured on 5% sheep blood-enriched Columbia agar (COS, BioMérieux, Marcy l’Etoile, France). After 18 h of incubation at 37°C, colonies were removed and suspended in NaCl at the required concentration. Three different strains of each species were used, thus Methicillin sensitive *S. aureus* (P6142, P2188 and P6141) and *S. sanguinis* (P8633, P760 and P2754). All strains were isolated from positive blood cultures.

### Light Transmission Aggregometry (LTA)

Platelet aggregation was analyzed by a turbidimetric method with a lumi-Aggregometer (APACT-4004, Elitech, France) (Chia et al., 2004). PPP was used to adjust 100% aggregation” and PRP was used to adjust the baseline. PRP was prewarmed for 3 min prior to the addition of bacteria, all the procedure was carried out at 37°C with shaking at 900 rpm. 20 µl of bacterial suspension were added to 180 µl of PRP. Bacteria concentrations have been previously optimized. Indeed, *S. aureus* strains were added from initial suspension of 10^9^ CFU/ml (Arman et al., 2014; Hannachi et al., 2019a) while *S. sanguinis* strains were added from initial suspension of 3 · 10^9^ CFU/ml to reach a final bacterial concentration in PRP equivalent to 10^8^ CFU/ml and 3 · 10^8^ CFU/ml respectively. The reaction had proceeded for at least 20 min, and the degree of aggregation was expressed as a percentage of aggregation (Light transmission before the addition of bacteria—light transmission after the addition of bacteria) x 100. Untreated PRP supplemented by 20 µL of NaCl or 10 µM of *Thrombin* Receptor-Activating Peptide (TRAP) (STAGO**^®^**, France) were used as negative and positive controls respectively.

### Analysis of Platelet Activation by Flow Cytometry

About 180 µl of treated or untreated platelets (250 G/L), as described above, were incubated with 20 µl of *S. aureus* P6142 or *S. sanguinis* P8633 (10^9^ CFU) (strains selected randomly). NaCl and TRAP (10 µM) were used as controls. Then, 4 μl of Phycoerethrin/Cy5 Anti CD62P antibody (IgG,ĸ monoclonal, BD Biosciences, San Jose, CA, USA) was added to 50 μl of sample and vortexed. Samples were incubated at room temperature in the dark for 30 min, then, 200 μl of Tyrode’ buffer was added before analysis by flow cytometer (Beckman Coulter FC500, Fullerton, CA, USA). Mean fluorescence intensity (MFI) of untreated, uninfected platelets was expressed as 100%. MFI for the other conditions was calculated as follow: MFI × 100/MFI of untreated, uninfected platelets.

### Scanning Electron Microscopy

Some 180 µl of treated and untreated PRP (250 G/L) as described above were incubated with 20 µl of *S. aureus* P6142 or *S. sanguinis* P8633 (10^9^ CFU/ml) for 20 min at 37°C under rotation to avoid the static state and the development of aggregates related to gravity. Cells were fixed using 2.5% glutaraldehyde in 0.1 M sodium cacodylate buffer for 1 h. Then, samples were rinsed three times with 0.1 M sodium cacodylate (5 min each) to remove residual fixative. Cells were dehydrated with graded ethanol concentrations: 25% for 5 min; 50% for 5 min; 70% for 5 min; 85% for 5 min; 95% for 5 min (twice); 100% ethanol for 10 min (three times). After ethanol dehydration, cells were incubated for 5 min in an ethanol/HMDS (1:2) mixture, then two times in pure HMDS. Between all steps, cells were gently stirred and centrifuged at 1,300*g*.min^−1^. A drop of cells in pure HDMS was deposited on a glass slide and allowed to air dry for 30 min before observation (Dukes et al., 2011). Cells were visualized under a TM4000Plus^®^ (Hitachi, Japan) scanning electron microscope operated at 10 and 15 kV in BSE mode at magnifications ranging from ×200 to ×3,000.

### Statistical Analysis

Statistical analysis was performed using GraphPad Prism for Windows. Significant differences between two groups were determined using the two-tailed, paired student’s *t* test. All other group comparisons were analyzed using one-way ANOVA with Bonferroni’s multiple comparison test. Statistical significance was set at p <0.05.

## Results

### Antiplatelet Drugs Showed Distinct Efficacy on Inhibiting Bacterial-Induced Platelet Aggregation

#### Ticagrelor Exhibited the Greatest Inhibitory Effect on Platelet Aggregation Induced by *S. aureus*

**Figure 1:**
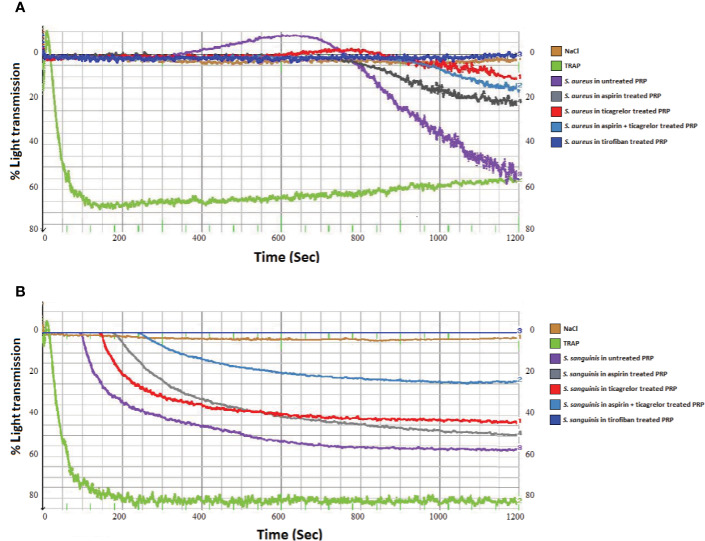
Representative measurement of light transmission over time in response to platelet aggregation. Untreated and aspirin (2 mM), ticagrelor (10 µM), association of both and tirofiban (25 ng/ml) treated PRP, infected by **(A)** *S. aureus* P2188 or **(B)** *S. sanguinis* P8633. NaCl and TRAP (10 µM) treated PRP were used as controls.

**Figure 2:**
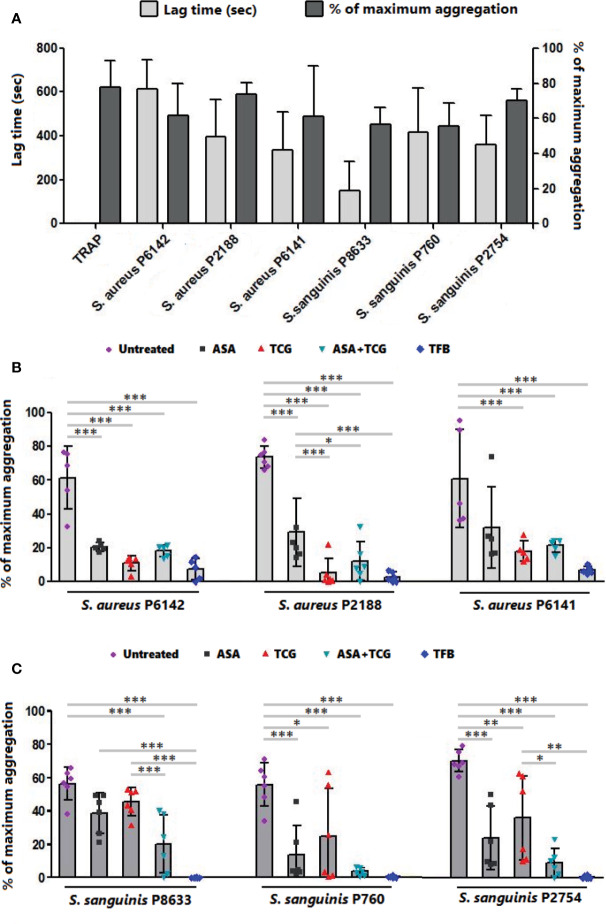
Effect of antiplatelet drugs on bacterial induced platelet aggregation. **(A)** Lag time for the onset of aggregation and percentage of maximum aggregation as measured by LTA of PRP activated by TRAP (10 µM) or infected with six bacterial strains belonging to two species, *S. aureus* and *S. sanguinis*. Results are expressed as mean ± SD. **(B)** Final aggregation as measured by LTA of untreated PRP and PRP treated with aspirin (Asp, 2 mM), ticagrelor (Tcg, 10 µM), association of both (Asp + Tcg) and tirofiban (Tfb, 0.5 µM) and then infected by three different strains of *S. aureus*. Results are expressed as mean ± SD. n = 5–6; one-way ANOVA, Bonferroni’s multiple comparison test. *: p < 0.05; **: p < 0.01; ***: p < 0.001. **(C)** Final aggregation as measured by LTA of untreated PRP and PRP treated with aspirin (Asp, 2 mM), ticagrelor (Tcg, 10 µM), association of both (Asp + Tcg) and tirofiban (Tfb, 0.5 µM) and then infected by three different strains of *S. sanguinis*. Results are expressed as mean ± SD. n = 6; one-way ANOVA, Bonferroni’s multiple comparison test. *: p < 0.05; **: p < 0.01; ***: p <0.001.

As observed in **Figures 1A** and **2A**, incubation of PRP with each of the three strains of *S. aureus* induced platelet aggregation, with a lag time that varied from 220 to 615 s according to the strain. While pretreatment of PRP with aspirin decreased platelet aggregation compared to untreated PRP, whatever the strain, the greatest decrease has been observed when PRP was pretreated with ticagrelor (**Figures 1A** and **2B**). A significant difference between aspirin and ticagrelor was obtained with the strain P 2188. Surprisingly, pretreatment of platelets with the combination, aspirin-ticagrelor, did not showed any additional or synergistic effect. As expected, tirofiban totally suppressed the aggregation.

#### Aspirin and Ticagrelor Combined Provided the Greatest Inhibitory Effect on Platelet Aggregation Induced by *S. sanguinis*

Incubation of PRP with *S. sanguinis*, whatever the strain tested, resulted in a strong platelet aggregation, with a lag time varying from 150 to 420 s according to the strain (**Figures 1B** and **2A**). Although aspirin and ticagrelor used separately slightly decreased platelet aggregation compared to untreated and infected PRP (**Figures 1B** and **2C**), the highest decrease was obtained when PRP was pretreated with the association of the two drugs. Significant differences were obtained between ticagrelor and the association with strains P8633 and P2754. As observed with *S. aureus*, Tirofiban showed complete inhibition of platelet aggregation induced by *S. sanguinis*.

### Antiplatelet Drugs Showed Distinct Effect on Platelet Activation Induced by Bacteria

**Figure 3:**
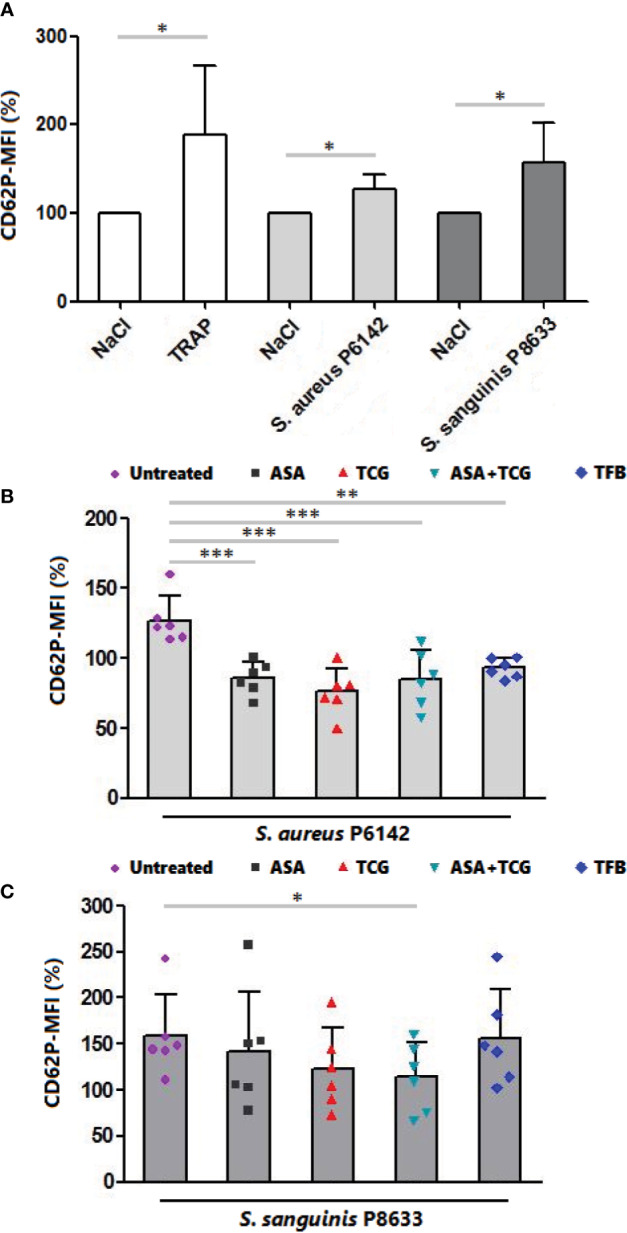
Measurement of CD62P surface exposure by flow cytometry. **(A)** Surface exposure of CD62P on native platelets, platelets activated by TRAP (10 µM) and platelets infected with *S. aureus* P6142 or *S. sanguinis* P8633. The MFI of untreated platelets was used as 100%. The MFI of each experiment was calculated as follow: MFI × 100/MFI of untreated and uninfected platelets. Results are expressed as mean ± SD. n = 6; Paired t test. *: p < 0.05. **(B)** Surface exposure of CD62P on untreated platelets and platelets treated with aspirin (Asp, 2 mM), ticagrelor (Tcg, 10 µM), association of both (Asp + Tcg) and tirofiban (Tfb, 0.5 µM). Untreated or treated platelets were then infected with *S. aureus* P 6142. The MFI of untreated platelets was used as 100%. The MFI of each experiment was calculated as follow: MFI × 100/MFI of untreated and uninfected platelets. Results are expressed as mean ± SD. n = 6; one-way ANOVA, Bonferroni’s multiple comparison test. **: p < 0.01; ***: p < 0.001. **(C)** Surface exposure of CD62P on untreated platelets and platelets treated with aspirin (Asp, 2 mM), ticagrelor (Tcg, 10 µM), association of both (Asp + Tcg) and tirofiban (Tfb, 0.5 µM). Untreated or treated platelets were then infected with *S. sanguinis* P 8633. The MFI of untreated platelets was used as 100%. The MFI of each experiment was calculated as follow: MFI × 100/MFI of untreated and uninfected platelets. Results are expressed as mean ± SD. n = 6; one-way ANOVA, Bonferroni’s multiple comparison test. *: p < 0.05.

Evaluation of platelet activation using flow cytometry showed that platelet exposure to *S. aureus* P6142 induced a significant increase in CD62P (P-selectin) surface exposure compared to uninfected platelets (n = 6, p = 0.0178) (**Figure 3A**). The pretreatment of platelets with anti-platelet drugs before exposure to *S. aureus* significantly reduced the mean intensity of CD62P surface exposure compared to untreated platelets, incubated with the same strain. Although not significant compared to the other treatments, ticagrelor was accompanied by the minimal surface exposure of CD62P on platelets (**Figure 3B**).

Regarding *S. sanguinis*, incubation of platelets with *S. sanguinis* P8633 induced also platelet activation as observed through the significant increase in CD62P surface exposure compared to uninfected platelets (n = 6, p = 0.0281) (**Figure 3A**). Using drug treated PRP, only the combination, aspirin and ticagrelor, showed a significant decrease compared to untreated platelets incubated with the same strain. Surprisingly, Tirofiban did not decrease platelet activation induced by *S. sanguinis* (**Figure 3C**).

Without bacteria, treatment of platelets resulted on a non-significant decrease of platelet CD62P surface exposure compared to untreated platelets (**Supplementary Figure 1**).

### The Ultrastructure of Platelet–Bacteria Aggregates Presented Major Differences According to the Inductive Bacterial Species

**Figure 4:**
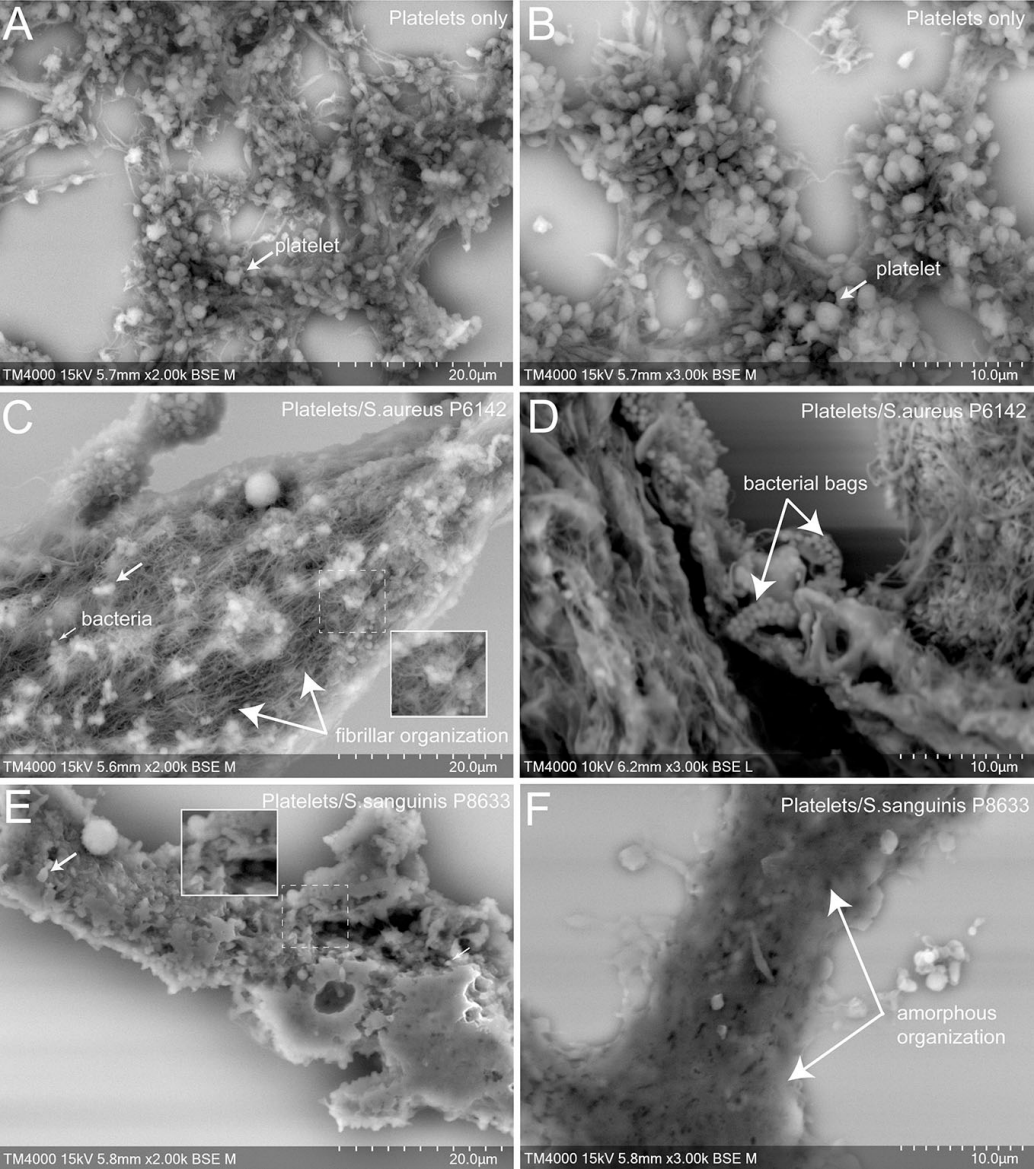
Scanning electron microscopy of uninfected platelets and platelets–bacteria aggregates. The whole deposited glass slide was analyzed for each condition with a TM4000Plus™ (Hitachi, Tokyo, Japan) scanning electron microscope operated at 10 and 15 kV in BSE mode at magnifications ranging from ×200 to ×3,000. Compared to uninfected control PRP **(A, B)**, the addition of *S. aureus* P6142 induced a change in the ultrastructure with a denser organization and the presence of an intense network with fibrillar organization. Also, several well-defined bags of bacteria were observed **(C, D)**. Clots obtained from PRP infected with *S. sanguinis* P8633 was organized in a form of a compact pack of amorphous clusters until an absence of cellular integrity **(E, F)**.

**Figure 5:**
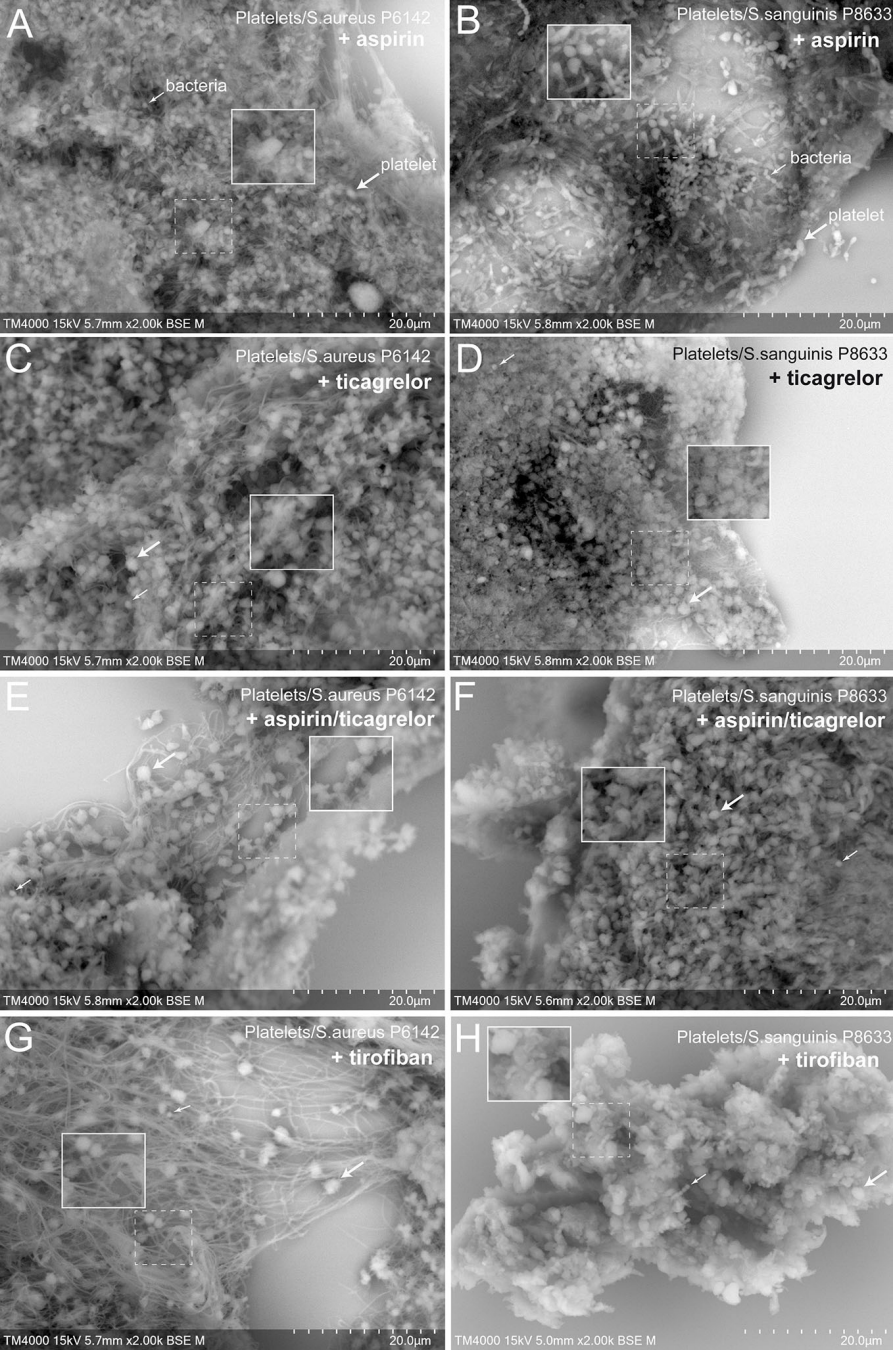
Scanning electron microscopy of aggregates formed from treated platelets and infected with *S. aureus* P6142 or *S. sanguinis* P8633. Aspirin treated PRP and infected with **(A)** *S. aureus* or **(B)** *S. sanguinis*. Ticagrelor treated PRP and infected with **(C)** *S. aureus* or **(D)** *S. sanguinis*. Aspirin-ticagrelor combination treated PRP and infected with **(E)** *S. aureus* or **(F)** *S. sanguinis*. Tirofiban treated PRP and infected with **(G)** *S. aureus* or **(H)** *S. sanguinis*. The whole deposited glass slide was analyzed for each condition with a TM4000Plus™ (Hitachi, Tokyo, Japan) scanning electron microscope operated at 10 and 15 kV in BSE mode at magnifications ranging from ×200 to ×3,000. The use of different antiplatelet molecules was accompanied by a slight decrease in the filamentous networks as well as in the organization of bagged bacteria in the case of *S. aureus*. For *S. sanguinis*, the use of antiplatelet agents was accompanied by a decrease in clot density and the possibility of detecting cellular elements within it.

To better understand the cell organization in the aggregates formed with platelets alone or mixed with infectious bacteria, as well as the effect of antiplatelet drugs, scanning electron microscopy (SEM) was used (**Figures 4** and **5**).

First, we observed control aggregates formed only by platelets derived from healthy subject (**Figures 4A, B**). Platelets in this condition were mostly well distinguishable one from another with some cells presenting protrusions typical of activated platelets. For the same aggregate, the density of platelets varied from one region to another, ranging from packed to single and dispersed platelets, mainly at the periphery.

Secondly, we observed aggregates of platelets mixed with bacteria. Bacteria cocci appeared isolated or arranged in clusters located on the surface of the aggregates or deeper between platelets. Platelets were more packed in the aggregates compared to the control, with a loss of cellular integrity. With *S. aureus* P6142, cells were condensed compared to control, with the presence of an intense network with fibrillar aspect. Also, several well-defined bags of bacteria were observed (**Figures 4C, D**). With *S. sanguinis* P8633, the increase of aggregate density was more pronounced, with aggregates mainly organized as a compact amorphous cell cluster (**Figures 4E, F**).

Finally, we observed aggregates of platelet pretreated with antiplatelet drugs and mixed with bacteria (**Figure 5**). Compared to untreated platelets, mixed with the same strains, the different antiplatelet agents exhibited no major differences in aggregates ultrastructures. However, antiplatelet drugs induced generally a decrease in the density of aggregates. We noticed a decrease in the fibrillar network and an absence of the bacterial bag organization in the case of *S. aureus* (**Figures 5A, C, E, G**). For *S. sanguinis*, platelets were well distinguishable in the aggregates, with preserved contour compared to untreated PRP (**Figures 5B, D, F, H**).

## Discussion

**Figure 6:**
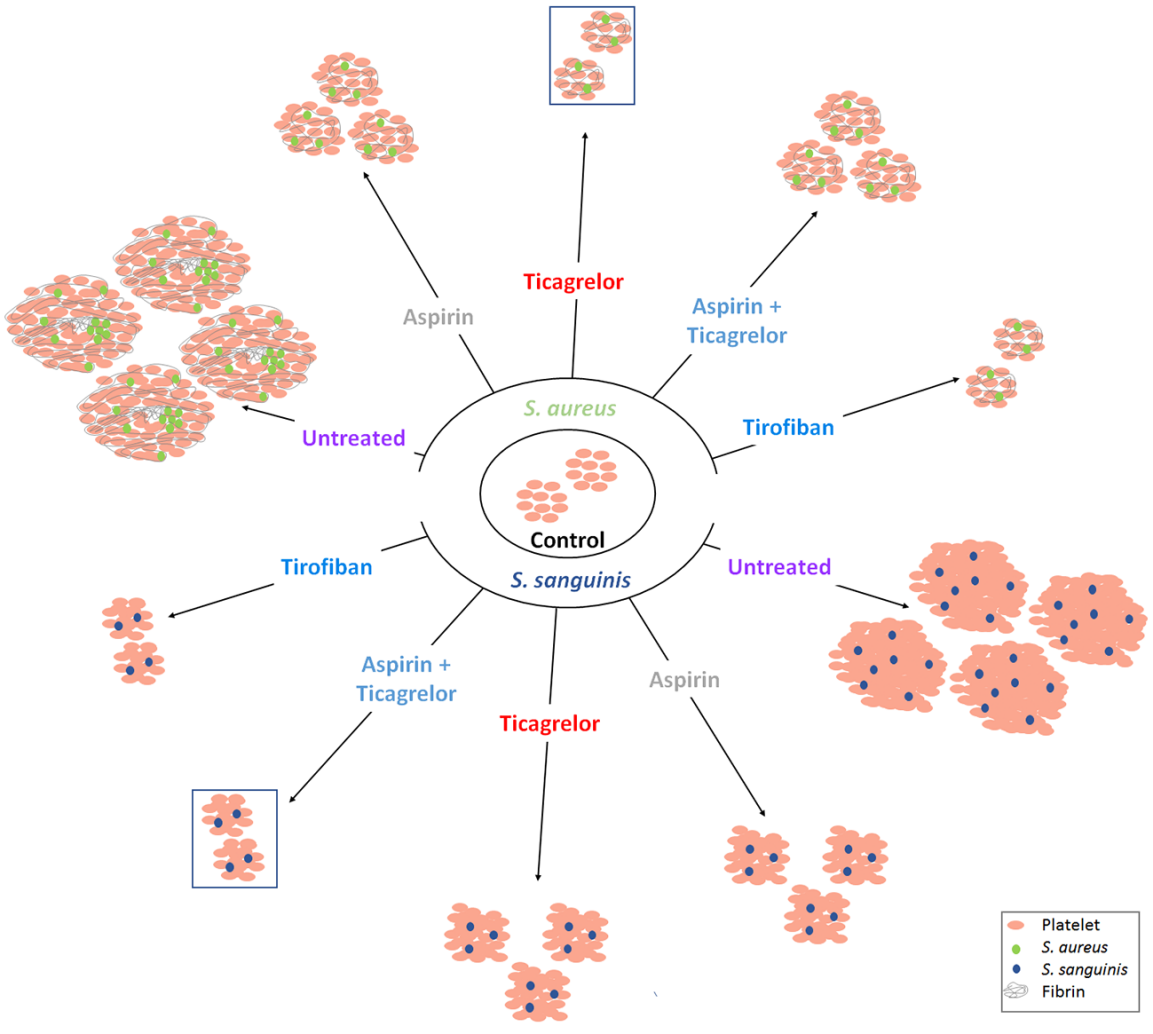
Interpretative scheme. Summarized results show here the effects of bacteria with or without antiplatelet drugs on platelets aggregation. Results of LTA and scanning electron microscopy are illustrated by three parameters: number of aggregates, their size and their ultrastructure. Antiplatelet agents significantly reduced aggregation induced by the two bacterial species. Among the antiplatelet agents taken orally and for long term, ticagrelor and the combination (aspirin–ticagrelor) showed the greatest decrease with *S. aureus* and *S. sanguinis* respectively (framed aggregates). The ultrastructure was different according to the two species tested.

All bacterial strains used in this study belong to two species most involved in IE and known to induce platelet activation and aggregation (Cox et al., 2011). To the best of our knowledge, we reported, for the first time, the differential efficacy of each of the antiplatelet drugs according to the bacterial species involved. Ticagrelor exhibited the greatest inhibitory effect on platelet aggregation in response to *S. aureus* while the association of aspirin and ticagrelor exhibited the greatest inhibitory effect in the case of *S. sanguinis*, regardless of the involved strain of each species. Our results are summarized in **Figure 6**, illustrating the overall aggregation and scanning electron microscopy results for each tested condition.

Our results regarding the effect of aspirin on platelet aggregation induced by *S. aureus* are consistent with those of a previous study, in which, the authors used indomethacin, another cyclo-oxygenase inhibitor and which showed a significant decrease in platelet aggregation (Arman et al., 2014). This benefit has also been confirmed in our recent study (Hannachi et al., 2019a), and in an animal model of IE (Veloso et al., 2015). In our experiments here, we focused on evaluating the effect of aspirin on platelet cyclooxygenase inhibition. However, aspirin metabolite, salicylic acid (SAL), has also been shown to influence platelet–*S. aureus* interactions by acting on gene regulation of bacterial virulence factors (Kupferwasser et al., 2003). Indeed, SAL has been shown to decrease, inter alia, the gene expression of several staphylococcal adherent motifs as well as staphylococcal alpha toxin (Kupferwasser et al., 2003; Herrmann, 2003; Hannachi et al., 2019a), both involved in platelet aggregation (Surewaard et al., 2018).

Despite a significant inhibition obtained with aspirin in the case of *S. aureus*, the strongest effect was obtained using ticagrelor. Similar results were reported by Arman et al. (2014) using cangrelor, an intravenous inhibitor of P2Y_12_ with short half-life. To the best of our knowledge, our study is the first to demonstrate the benefit of an oral anti P2Y_12_ receptor in platelet aggregation induced by bacteria. This result might highlight the key role of ADP pathway in mediating platelet aggregation induced by *S. aureus*. In parallel, and far from platelet aggregation, Lancellotti et al. have recently reported a bactericidal activity of ticagrelor against *S. aureus* (Lancellotti et al., 2019).

Regarding the use of antiplatelets with *S. sanguinis*, conflicting results have been reported. It has been shown that platelet activation induced by *S. sanguinis* involves both cyclooxygenase and ADP pathways (MacFarlane et al., 1994; Cox et al., 2011). Ford et al. reported an abolishment of platelet aggregation by aspirin (Ford et al., 1993). By opposite, we and others have previously shown no significant effect using only cyclooxygenase pathway inhibitor (Arman et al., 2014; Hannachi et al., 2019a). In this current study, by testing different strains, we reported a variable inhibition of platelet aggregation using aspirin according to each strain. This result could explain the discrepancy between the studies mentioned above. Despite this inhibitory effect of aspirin, it was always below to that achieved by blocking both cyclooxygenase and ADP pathways simultaneously. Contrary to expectations, we underlined here that the expression of this synergistic effect depended on the bacterial species involved, as was the case in presence of *S. sanguinis*, but not with *S. aureus* (Arman et al., 2014; Veloso et al., 2015).

Regarding the effect of tirofiban, we reported an inhibition of both platelet activation and aggregation induced by *S. aureus*. Similar results have been previously reported (Herrmann, 2003). The GP IIb IIIa is considered the main glycoprotein implied in the interaction of platelets with *S. aureus via* the Iron-regulate surface determinant B (Isd B) and clumping factor (Clf) A and B expressed on the surface of the latter (Miajlovic et al., 2010). In the case of *S. sanguinis*, tirofiban resulted in an inhibition of platelet aggregation without effect on CD62P surface exposure. A similar result was previously observed by Kerrigan et al., where GP IIb IIIa receptor antagonism resulted in the inhibition of platelet aggregation by *S. sanguinis* without effect on the adhesion of the bacteria to the platelets (Kerrigan et al., 2002). This suggest that in case of *S. sanguinis* infection, GP IIbIIIa inhibition prevents platelet aggregation without action on their individual activation induced by the bacteria.

Scanning electron microscopy allowed us to analyze qualitatively the ultrastructure of the platelet-bacteria aggregates. Platelet arrangement was different according to the incriminated bacterial species, with a dense organization of the aggregate as an amorphous cluster and a loss of cellular integrity in case of *S. sanguinis*, and a presence of bacterial bags and a large network of filaments in the case of *S. aureus*. These filaments might be a fibrin network (Gersh et al., 2009) explained by the ability of *S. aureus* to trigger the coagulation step *via* its two coagulases: staphylo-coagulase (Coa) and von Willebrand factor binding protein (VWbp) (Bjerketorp et al., 2004; Thomer et al., 2013). In our previous study, we made similar observations with untreated washed platelets incubated with the same species (Hannachi et al., 2019a). The treatment of PRP with antiplatelet drugs induced a decrease in the aggregate density in the case of *S. sanguinis* and an absence of bacterial bags and a decrease in the fibrin network in the case of *S. aureus*. However, for the last observation, despite this decrease linked to platelet inhibition, it was not total. This persistence of the filamentous network may be linked to the capacity of *S. aureus* coagulases to bypass the primary hemostasis step and therefore keeping the possibility of fibrin formation despite platelet inhibition.

Our study may explain the discrepancy between previous clinical studies interested in the benefit of antiplatelet drugs on infectious diseases with thrombotic events (Chan et al., 2003; Anavekar et al., 2007; Pepin et al., 2009), where statistical analyzes were performed independently on the bacterial species. Moreover, we demonstrated an inter strain discrepancy was noted in this study. Also, we might recommend considering the involved bacterial strain for optimal antiplatelet therapy in clinical practice. Aggregometry technique could serve as an easy and quick routine test to screen for the best antiplatelet agent using the patient’s platelets as well as the isolated bacterial strain.

In our study, we used an *in vitro* model focusing on the effect of antiplatelet agents on the platelet aggregation induced by bacteria. However, we are aware that evaluating the effect of these drugs considering other types of human cells, such as neutrophils and endothelial cells, in addition to platelets, may provide more information and better relate to *in vivo* conditions (Lubkin and Torres, 2016; Hannachi et al., 2019b). Further experimental and clinical studies are required to elucidate the distinct effects of antiplatelet drugs in the management of diseases related to bacterial-induced platelet aggregation, allowing targeted-antiplatelet treatment to be provided to selected patients and specific bacterial strains.

## Data Availability Statement

All datasets generated for this study are included in the article/**Supplementary Material**.

## Ethics Statement

This study was carried out in accordance with the recommendations of IHU Méditerranée-infection committee. The protocol was approved by the IHU Méditerranée-infection committee (reference 2016-002). All subjects gave written informed consent in accordance with the Declaration of Helsinki.

## Author Contributions

NH and LC-J designed the protocol, NH, EO-G, J-PB, AF, and DB performed experiments. NH, J-PB, GH, and LC-J performed analysis and wrote the manuscript.

## Funding

This work was supported by the Institut Hospitalo-Universitaire (IHU) Méditerranée-Infection, Marseille, France.

## Conflict of Interest

The authors declare that the research was conducted in the absence of any commercial or financial relationships that could be construed as a potential conflict of interest.

## Supplementary Material

Supplementary Figure 1 Measurement of CD62P surface exposure by flow cytometry. Surface exposure of CD62P on native platelets and platelets treated with antiplatelets drugs (Aspirin, ticagrelor, aspirin-ticagrelor and tirofiban). The MFI of untreated platelets was used as 100%. The MFI of each experiment was calculated as follow: MFI x 100/MFI of untreated and uninfected platelets. Results are expressed as mean ± SD.
