# Supplementary figures and images for: Antiplatelet Agents Have a Distinct Efficacy on Platelet Aggregation Induced by Infectious Bacteria
Source: Front Pharmacol. 2020 Jun 5;11:863. doi: 10.3389/fphar.2020.00863 (PMC7291881; doi:10.3389/fphar.2020.00863)

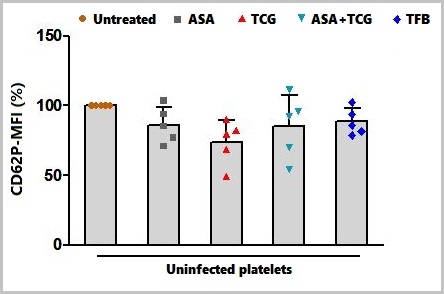

Supplement: Supplementary Figure 1 — Measurement of CD62P surface exposure by flow cytometry. Surface exposure of CD62P on native platelets and platelets treated with antiplatelets drugs (Aspirin, ticagrelor, aspirin-ticagrelor and tirofiban). The MFI of untreated platelets was used as 100%. The MFI of each experiment was calculated as follow: MFI x 100/MFI of untreated and uninfected platelets. Results are expressed as mean ± SD. [file Image_1.tif]
